# Oral sulfate solution benefits polyp and adenoma detection during colonoscopy: Meta‐analysis of randomized controlled trials

**DOI:** 10.1111/den.14299

**Published:** 2022-04-06

**Authors:** Cheng Chen, Mengyang Shi, Zhongli Liao, Weiqing Chen, Yongzhong Wu, Xu Tian

**Affiliations:** ^1^ Department of Gastroenterology, Chongqing Key Laboratory of Translational Research for Cancer Metastasis and Individualized Treatment Chongqing University Cancer Hospital Chongqing China; ^2^ 605425 Radiation Oncology Center Chongqing University Cancer Hospital Chongqing China; ^3^ Nursing Department Universitat Rovira I Virgili Tarragona Spain

**Keywords:** adenoma, bowel preparation, colonoscopy, colorectal cancer, oral sulfate solution

## Abstract

**Objectives:**

Although oral sulfate solution (OSS) has been revealed to be not only safe and efficacious but also noninferior to polyethylene glycol with ascorbic acid (PEG + ASC), it is unclear whether OSS can ultimately increase the polyp detection rate (PDR) and adenoma detection rate (ADR). We performed this meta‐analysis to estimate the effect of OSS on PDR and ADR during colonoscopy.

**Methods:**

We searched PubMed, EMBASE, and the Cochrane Library to identify relevant randomized controlled trials (RCTs) investigating the comparative effect of OSS versus PEG + ASC on the PDR and ADR during colonoscopy. Cecal intubation time (CIT), cecal intubation rate (CIR), and bowel preparation score were also evaluated. Review Manager (RevMan) version 5.3.0 was used to perform statistical analysis.

**Results:**

Eight RCTs involving 2059 patients fulfilled the selection criteria. Meta‐analysis suggested that OSS significantly increased the PDR (47.34% vs. 40.14%, risk ratio [RR] 1.13, 95% confidence interval [CI] 1.03−1.24, *P* = 0.01) and ADR (44.60% vs. 38.14%, RR 1.17, 95% CI 1.03−1.33, *P* = 0.01) during colonoscopy. Subgroup analysis showed that the beneficial effects of OSS on PDR and ADR were consistent among patients with mean age >55 years and with body mass index <25 kg/m^2^ receiving outpatient colonoscopy, morning colonoscopy, and the 2‐L bowel preparation protocol. Meanwhile, patients receiving OSS had a beneficial bowel preparation score.

**Conclusion:**

Compared with polyethylene glycol‐based regimens, the OSS bowel preparation regimen significantly increased the PDR and ADR in patients undergoing colonoscopy.

## INTRODUCTION

Colonoscopy is considered the most common method in screening, diagnosis, and surveillance of colorectal cancer (CRC).^1^, ^2^ Sufficient bowel preparation is essential to perform high‐quality colonoscopy.^3^, ^4^, ^5^, ^6^ Nonetheless, suboptimal bowel preparation accounts for approximately one‐quarter of failed colonoscopies.^7^ Insufficient bowel preparation can reduce the detection rate of polyp and adenoma,^8^ prolongs procedural duration,^9^ and increases the risk of colonoscopy‐associated complications,^10^ the need for a repeated colonoscopy,^11^ and medical expenditures.^12^

Polyethylene glycol (PEG) has been historically used as the criterion standard for bowel preparation;^10^, ^11^, ^13^ however, patients find it difficult to fully consume the bowel preparation solution due to the large intake volume and unpleasant palatability of the conventional 4‐L PEG solution.^14^ Therefore, low‐volume PEG with improved flavor and comparable efficacy and safety have been developed. Two‐liter PEG with additional ascorbic acid (PEG + ASC) has been developed and determined to be more tolerable than 4‐L PEG and have comparable efficacy and safety.^15^, ^16^, ^17^ Beyond that, another new low‐volume bowel preparation agent, oral sulfate solution (OSS), has also been developed for bowel cleansing in 2009,^18^ and its efficacy and safety has been reported in studies.^18^, ^19^

Concurrently, several randomized controlled trials (RCTs) have demonstrated that, compared with PEG‐based bowel preparation solutions, OSS has comparable or better efficacy and safety, as well as superior tolerability and acceptability.^20^, ^21^, ^22^ A recent meta‐analysis suggested that, compared with PEG plus ASC, an OSS regimen substantially increased the rate of excellent bowel preparation among individuals at low risk of inadequate bowel preparation;^23^ however, it is unclear whether OSS actually increases the detection of precursor polyps, CRC, or other lesions^24^ because the quality of bowel preparation is just a surrogate indicator for detection of colonic lesions. Therefore, the magnitude of benefit in detecting polyps and adenomas needs to further evaluated. Here we performed a meta‐analysis to evaluate the influence of the OSS regimen as a bowel preparation laxative regimenn on polyp detection rate (PDR), adenoma detection rate (ADR), and other outcomes.

## METHODS

The current META‐ANALYSIS was performed according to the Preferred Reporting Items for Systematic Reviews and Meta‐Analysis.^25^ No ethical approval or patients' informed consent was required because the statistical analysis was based on published data.

### Search strategies

Two investigators independently searched PubMed, EMBASE, and the Cochrane library to identify potentially relevant RCTs investigating the comparative effect of OSS versus PEG‐based solutions for bowel preparation on PDR and ADR during colonoscopy through October 2021. The following terms and their analogs were used to construct search strategies, including colonoscopy, bowel cleansing, and oral sulfate solution. Additionally, we also checked all the references of topic‐related reviews and included RCTs in order to add relevant studies. Conference abstracts or unpublished reports were not considered. Any discrepancy between the two investigators was settled by consulting a third investigator. Detailed search strategies are summarized in Table S1.

### Study selection

Two investigators independently assessed all the potentially relevant studies according to the selection criteria as follows: (i) study participants were adult patients receiving colonoscopy; (ii) OSS used for bowel preparation in a study group and PEG‐based solutions used for bowel preparation in a control group; (iii) RCT reported with outcome measures including PDR, ADR, cecal intubation time (CIT), cecal intubation rate (CIR), and bowel preparation scores; and (iv) only studies published in the English language were considered. In this meta‐analysis, PDR and ADR were considered as the primary outcomes. CIT, CIR, and the bowel preparation score were considered the secondary outcomes. Bowel preparation scores were determined by using the Ottawa Bowel Preparation Scale (OBPS)^26^ or Boston Bowel Preparation Scale (BBPS).^27^ We excluded ineligible studies according to the following criteria: (i) ineligible design such as experimental studies, reviews and commentary; (ii) duplicate studies; and (iii) studies with insufficient data. Any discrepancy between the two investigators was solved by consulting a third investigator.

### Data extraction

Two independent investigators accurately extracted essential data, including the first author, year of publication, location, type of colonoscopy, setting of performing colonoscopy, indications of colonoscopy, intervention information, methods of intaking bowel preparation solution, sample size, mean age and body mass index (BMI) of participants, outcome measures, and detailed information of methodology. Any discrepancy between the two investigators was solved by consulting a third investigator.

### Quality assessment

We used the Cochrane risk of bias assessment tool to evaluate the methodological quality of each eligible study^28^ from the following seven items, including random sequence generation, allocation concealment, blinding of participants and personnel, blinding of outcome assessor, incomplete outcome data, selective outcome reporting, and other sources. For each item, a label of low, unclear, or high risk of bias was granted according to the overlapping level between actual information in individual study and assessment criteria. Performance bias was considered as a low risk if both investigators and participants were blinded, as unclear risk if only the investigator or participants was blinded, or as high risk if both investigators and participants were not blinded. Detection bias was considered as low risk if the outcome assessment was performed by a blinded investigator because the primary outcomes were objective variables according to the criteria defined by a previous meta‐analysis.^29^

### Statistical analysis

Meta‐analysis was performed using Review Manager (RevMan, v. 5.3) (The Nordic Cochrane Centre, The Cochrane Collaboration, Copenhagen, Denmark).^30^ Statistical heterogeneity across studies was tested using the Cochrane *Q* ^31^ and the *I* ^2^ statistic.^32^ However, we used the random‐effects model to perform pooled estimate analysis because this model simultaneously incorporates variations between and within studies.^33^, ^34^ Pooled results of dichotomous variables were expressed as the risk ratio (RR) with the corresponding 95% confidence interval (CI), and pooled results of continuous variables were expressed as the mean difference (MD) with corresponding 95% CI. Difference was considered significant if *P* < 0.05. Moreover, we performed several subgroup analyses to identify potential differences among the trials to explore clinically significant heterogeneity and examine the robustness of the pooled results. We created funnel plots for primary outcomes to examine publication bias, although insufficient eligible studies were accumulated.^35^

## Results

### Study selection

**Figure 1:**
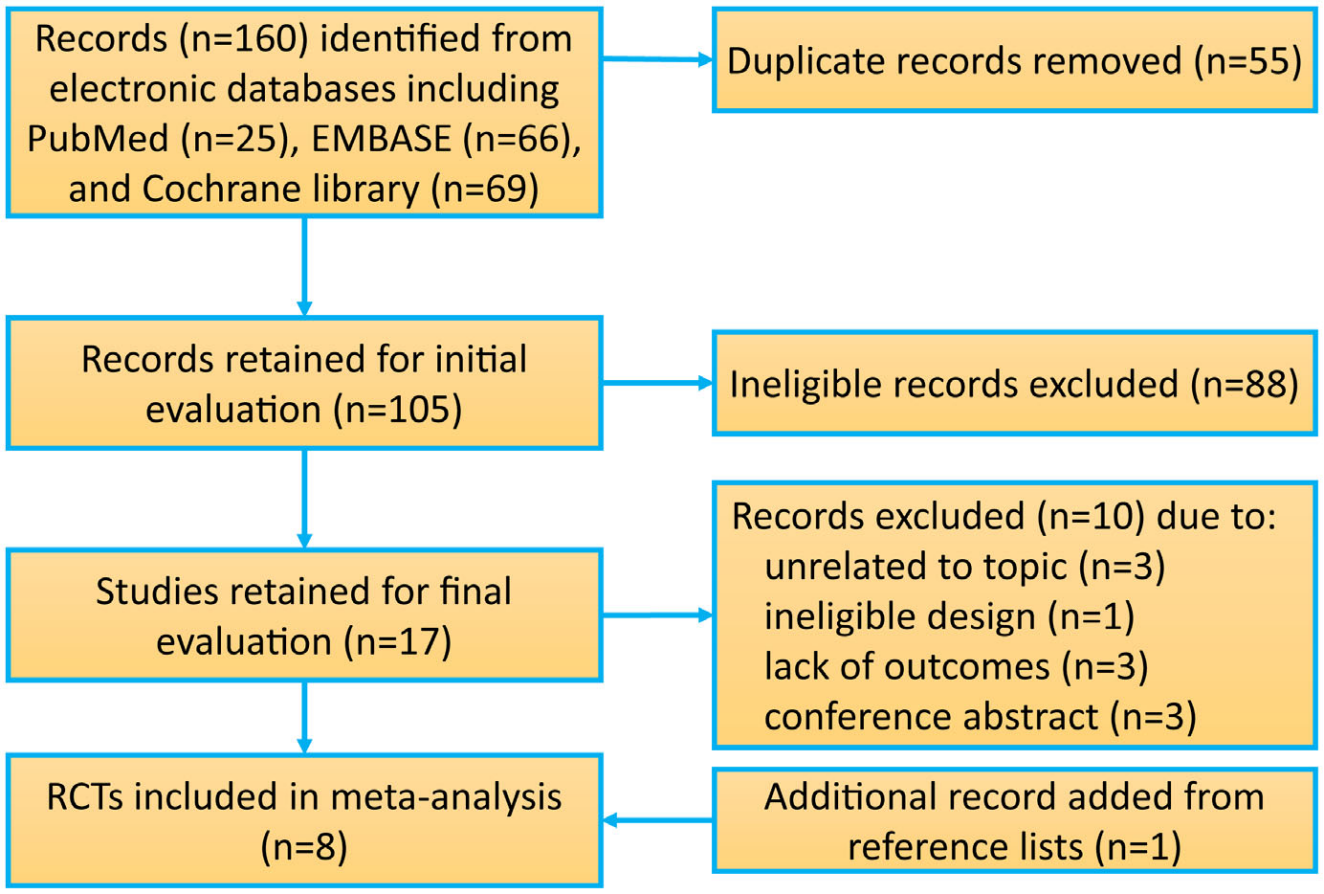
Flow diagram of retrieval and selection of relevant randomized controlled trials (RCTs).

Our search strategies initially identified 160 records from PubMed, EMBASE, and the Cochrane Library. A total of 55 duplicate records were removed based on software and 88 records were excluded based on titles and abstracts evaluation. Of the remaining 17 studies, 10 ineligible studies^14^, ^21^, ^22^, ^36^, ^37^, ^38^, ^39^, ^40^, ^41^, ^42^ were excluded based on full‐text evaluation for the following reasons: unrelated to topic (*n* = 3),^36^, ^38^, ^40^ ineligible design (*n* = 1),^41^ conference abstracts (*n* = 3),^37^, ^39^, ^42^ or lack of outcomes of interest (*n* = 3).^14^, ^21^, ^22^ Additionally, we identified another eligible study^43^ from published studies. Therefore, eight RCTs^20^, ^43^, ^44^, ^45^, ^46^, ^47^, ^48^, ^49^ were included for final analysis (Fig. 1).

### Study characteristics

All RCTs^20^, ^43^, ^44^, ^45^, ^46^, ^47^, ^48^, ^49^ were published between 2016 and 2021, and most RCTs^20^, ^44^, ^45^, ^46^, ^49^ were conducted in Korea. The sample size of all eligible RCTs ranged between 167 and 556, with a total sample size of 2059. Two RCTs^44^, ^47^ specifically enrolled elderly individuals, two RCTs^44^, ^49^ used high‐volume PEG‐based solution (4‐L PEG + ASC) and one RCT^43^ used ultra‐volume PEG‐based solution (1‐L PEG + ASC), three RCTs^20^, ^46^, ^47^ clearly stated to perform morning colonoscopy, and four RCTs^20^, ^46^, ^47^, ^48^ specifically considered outpatients. Characteristics of the eight RCTs are shown in Table 1. Specific data of primary and secondary outcomes of individual RCTs are summarized in Table 2.

**Table 1:** Characteristics of included studies (*n* = 8)

Study	Location	Type	Setting	Indications	Interventions		Regimen	Sample size		Mean age, years		Sex (male/female)		Mean BMI, kg/m^2^	
					PEG	OSS		PEG	OSS	PEG	OSS	PEG	OSS	PEG	OSS
Kim *et al*., 2017^20^	Korea	Morning colonoscopy	Outpatient	Screening surveillance	2 L PEG + ASC	2 L OSS	Split‐dose	84	83	57.1	55.7	46/38	48/35	23.9	23.8
Lee *et al*., 2019^46^	Korea	Morning and afternoon colonoscopy	Outpatient	Screening surveillance diagnostic treatment	2 L PEG + ASC	2 L OSS	Split‐dose	92	92	60	60.3	50/42	50/42	23.3	23.7
Nam *et al*., 2021^47^	South Korea	Morning colonoscopy	Outpatient	Screening surveillance diagnostic treatment	2 L PEG + ASC	2 L OSS	Split‐dose	94	95	72.1	70.9	68/26	48/47	n.r.	n.r.
Shah *et al*., 2019^48^	India	n.r.	Outpatient	Screening surveillance diagnostic treatment	2 L PEG + ASC	1 L OSS	Split‐dose	222	178	43.87	43.89	148/74	112/66	n.r.	n.r.
Yang *et al*., 2017^49^	Korea	Morning colonoscopy	Outpatient	Screening diagnostic treatment	4 L PEG + ASC	2 L OSS	Split‐dose	99	98	53.4	51.2	63/36	53/35	24	23.7
DeMicco *et al*., 2018^43^	USA	n.r.	Outpatient and inpatient	Screening surveillance diagnostic	1 L PEG + ASC	2 L OSS	Split‐dose	276	280	57.5	56.8	141/135	156/124	29.5	29.8
Kwak *et al*., 2019^44^	Korea	n.r.	n.r.	Screening surveillance diagnostic	4 L PEG + ASC	2 L OSS	Split‐dose	96	97	69.3	68.6	46/50	43/54	n.a.	n.a.
Kwon *et al*., 2020^45^	Korea	n.r.	n.r.	Screening	2 L PEG + ASC	2 L OSS	Split‐dose	87	86	56.2	53.6	39/48	37/49	23.9	23.7

ASC, ascorbic acid; BMI, body mass index; n.a., not applicable; n.r., not reported; OSS, oral sodium sulfate; PEG, polyethylene glycol.

**Table 2:** Outcomes of included studies

Study	PDR (%)		ADR (%)		OBPS		BBPS		CIT, min		CIR (%)	
	PEG	OSS	PEG	OSS	PEG	OSS	PEG	OSS	PEG	OSS	PEG	OSS
Kim *et al*., 2017^20^	38 (45.2)	50 (60.2)	27 (32.1)	35 (42.2)	5.0 ± 2.0	4.0 ± 2.0	n.a.	n.a.	n.a.	n.a.	n.a.	n.a.
Lee *et al*., 2019^46^	47 (51.1)	55 (59.8)	35 (38.0)	44 (47.8)	n.a.	n.a.	7.3 ± 1.2	7.4 ± 1.3	5.2 ± 4.5	5.6 ± 3.4	100	100
Nam *et al*., 2021^47^	66 (70.2)	72 (75.8)	51 (54.3)	65 (68.4)	n.a.	n.a.	7.69 ± 1.29	7.69 ± 1.57	5.6 ± 4.27	5.5 ± 3.05	98.9	98.9
Shah *et al*., 2019^48^	17 (7.7)	12 (6.7)	n.a.	n.a.	n.a.	n.a.	n.a.	n.a.	6.05 ± 4.1	5.23 ± 3.35	97.3	97.8
Yang *et al*., 2017^49^	54 (54.5)	66 (67.3)	40 (40.4)	44 (44.9)	n.a.	n.a.	7.9 ± 1.3	8.1 ± 1.3	4.7 ± 3.5	4.6 ± 2.7	100	100
DeMicco *et al*., 2018^43^	126 (45.7)	136 (48.6)	93 (33.7)	98 (35.0)	n.a.	n.a.	n.a.	n.a.	n.a.	n.a.	n.a.	n.a.
Kwak *et al*., 2019^44^	n.a.	n.a.	n.a.	n.a.	n.a.	n.a.	7.4 ± 1.3	7.9 ± 1.3	n.a.	n.a.	100	100
Kwon *et al*., 2020^45^	n.a.	n.a.	n.a.	n.a.	4.49 ± 3.08	2.8 ± 2.48	6.51 ± 1.76	7.43 ± 1.49	4.1 ± 2.68	4.0 ± 2.03	100	100

ADR, adenoma detection rate; BBPS, Boston Bowel Preparation Scale; CIR, cecal intubation rate; CIT, cecal insertion time; n.a., not available; OBPS, Ottawa Bowel Preparation Scale; OSS, oral sodium sulfate; PDR, polyp detection rate; PEG, polyethylene glycol.

### Quality assessment

**Figure 2:**
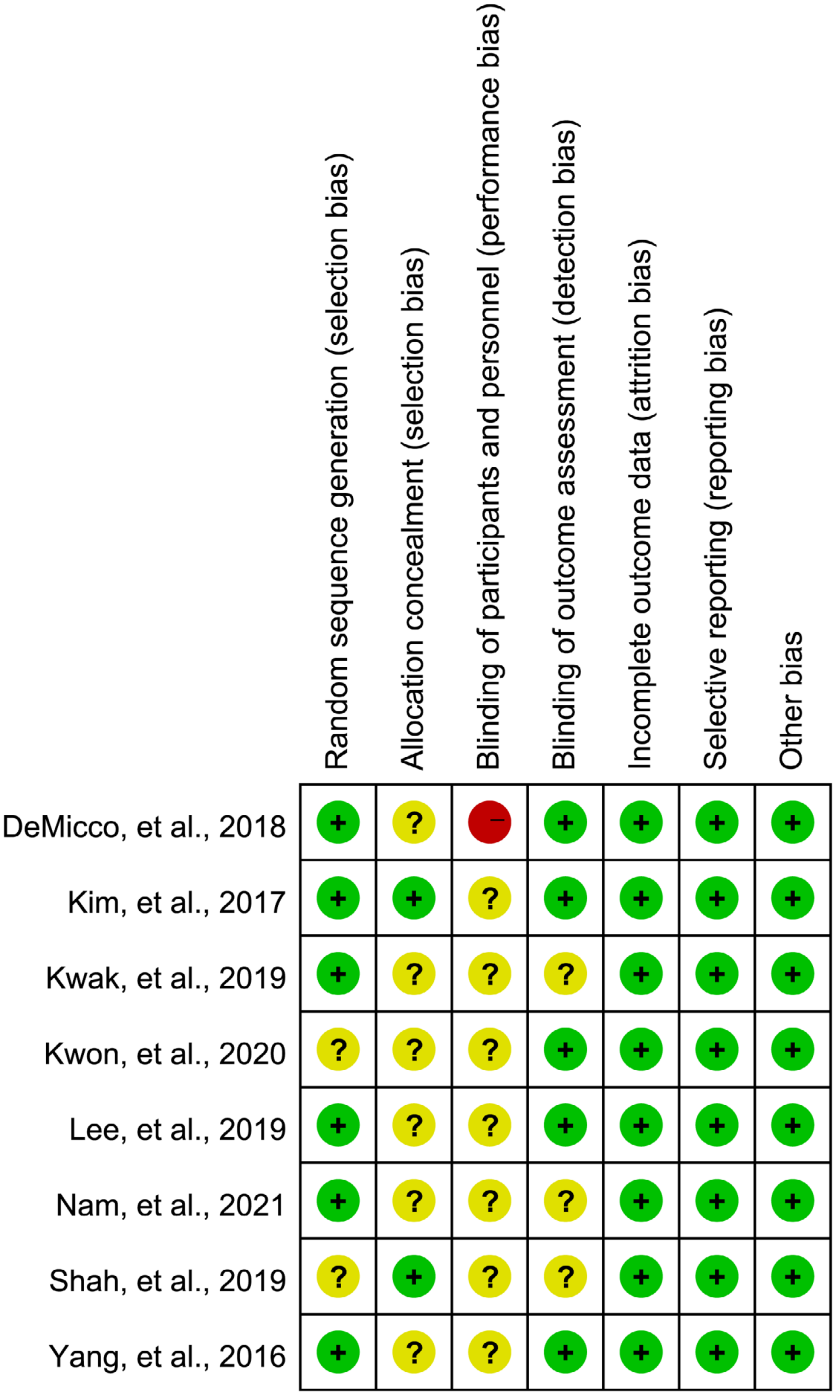
Risk of summary.

The methodological quality of individual RCTs is shown in Figure 2. Six RCTs^20^, ^43^, ^44^, ^46^, ^47^, ^49^ clearly reported the methods to generate a random sequence, but only two RCTs^20^, ^43^ clearly reported the approaches of concealing allocation. Seven studies^20^, ^44^, ^45^, ^46^, ^47^, ^48^, ^49^ blinded investigators but not participants and were therefore judged as unclear risk in performance bias except for one study,^43^ which did not blind either investigators or participants. Regarding outcome assessment, five studies^20^, ^43^, ^45^, ^46^, ^49^ were judged as low risk of bias because it was evaluated by ether blinded independent trained central readers or blind investigators; however, another three studies^44^, ^47^, ^48^ did not clearly describe detailed information on outcome assessment and were therefore rated as unclear risk. For the remaining items, all RCTs were considered as low risk.

### Primary outcomes and subgroup analysis

**Figure 3:**
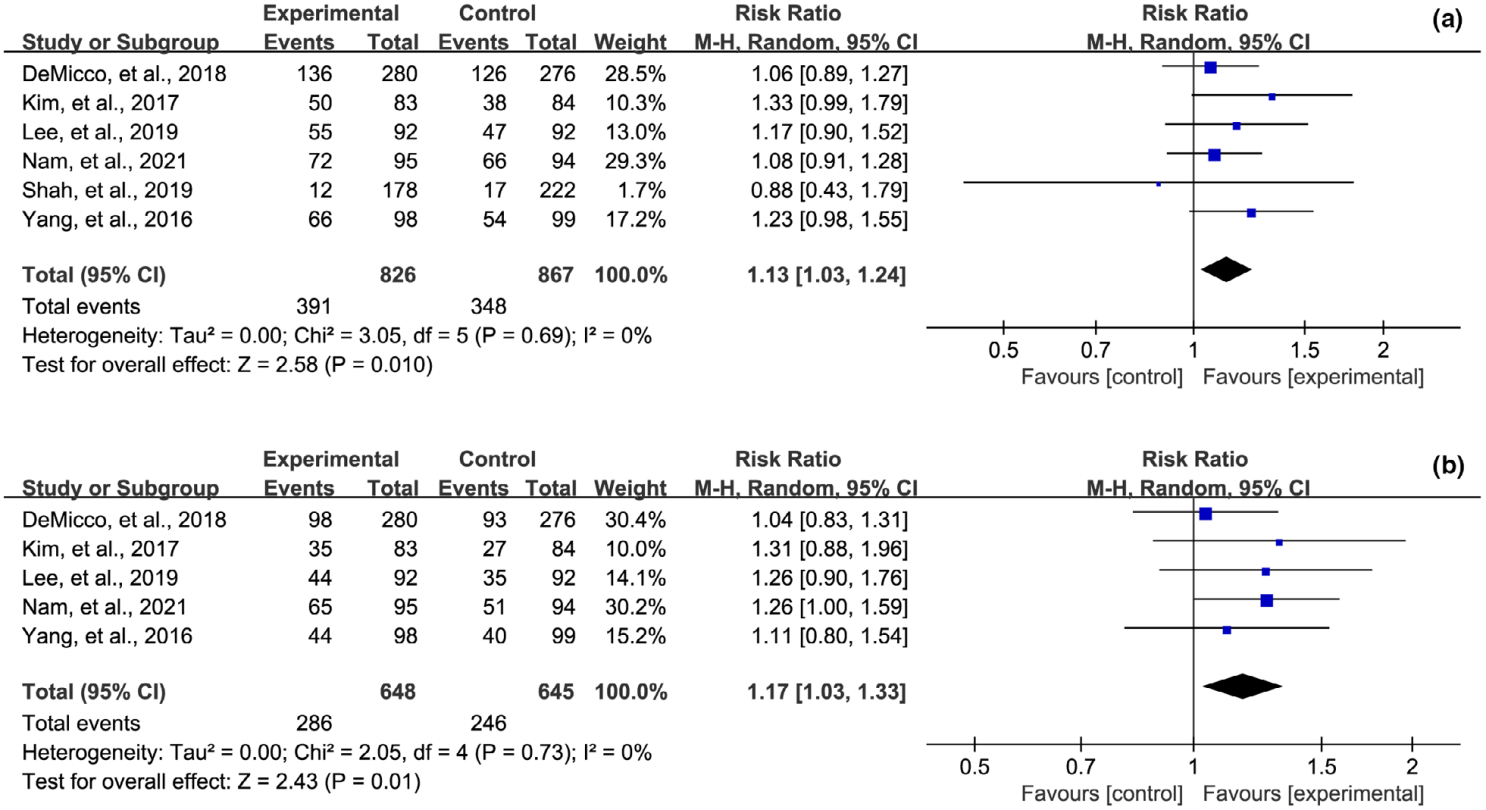
Meta‐analysis of the effect of oral sulfate solution on polyp (a) and adenoma (b) detection rate. CI, confidence interval; M‐H, Mantel−Haenszel.

As the primary outcome, PDR and ADR was reported in six^20^, ^43^, ^46^, ^47^, ^48^, ^49^ and five^20^, ^43^, ^46^, ^47^, ^49^ RCTs, respectively. The statistical heterogeneity in meta‐analysis of PDR or ADR had an *I* ^2^ value of 0% (PDR: *P* = 0.69; ADR: *P* = 0.73), suggesting no statistical heterogeneity. In the meta‐analysis of six RCTs that reported the PDR, the PDR of the OSS group was statistically higher than that of the PEG‐based solutions group (RR 1.13, 95% CI 1.03−1.24, *P* = 0.01), as shown in Figure 3a. In the meta‐analysis of five RCTs that reported ADR, the ADR of the OSS group was also statistically higher than that of the PEG‐based solutions group (RR 1.17, 95% CI  1.03−1.33, *P* = 0.01), as shown in Figure 3b. However, as shown in Figure S1, the pooled results of PDR and ADR might be negatively affected by the presence of publication bias.

**Figure 4:**
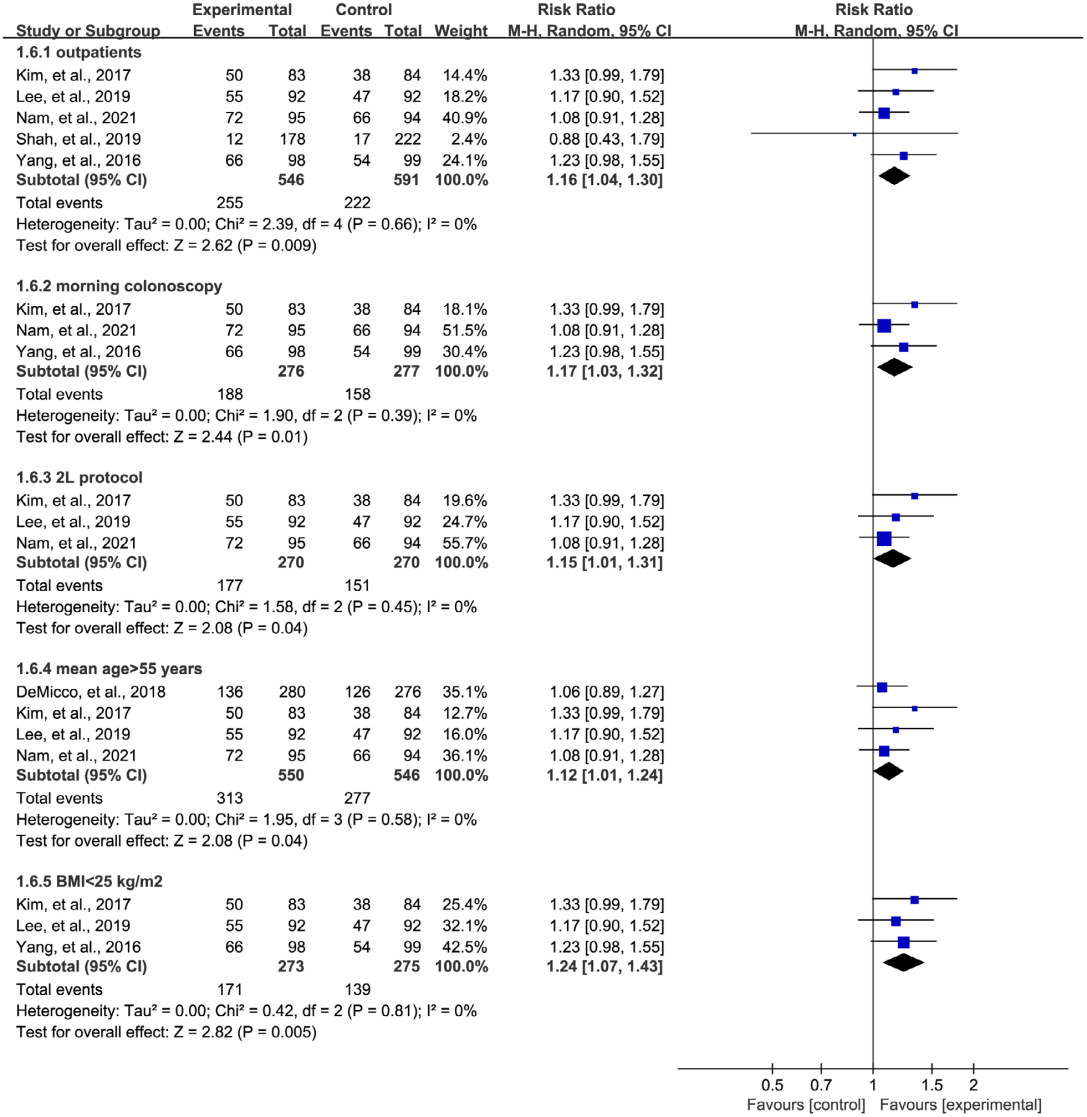
Subgroup analysis of the effect of oral sulfate solution on polyp detection rate in outpatients, morning colonoscopy, 2‐L bowel preparation protocols, patients with mean age of more than 55 years, and patients with body mass index (BMI) of less than 25 kg/m^2^. CI, confidence interval; M‐H, Mantel−Haenszel.

**Figure 5:**
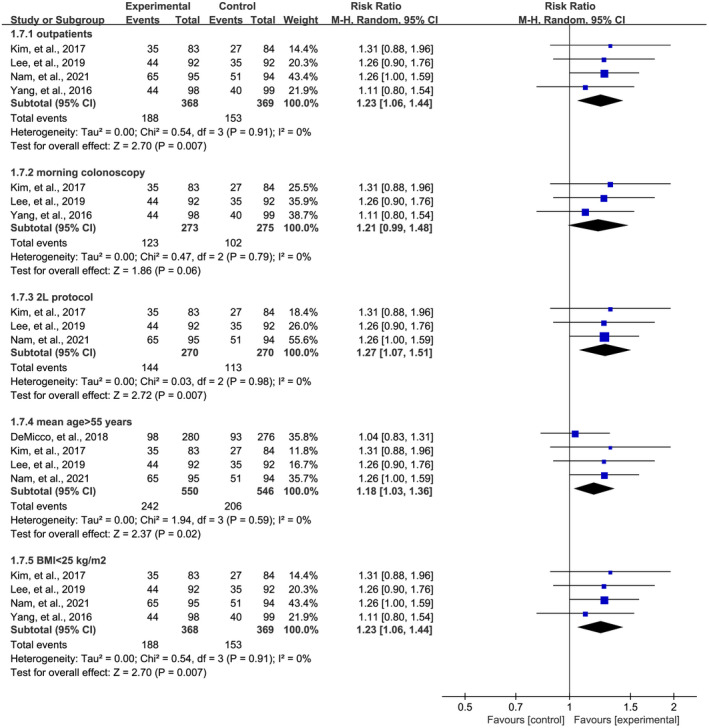
Subgroup analysis of the effect of oral sulfate solution on adenoma detection rate in outpatients, morning colonoscopy, 2‐L bowel preparation protocols, patients with mean age of more than 55 years, and patients with body mass index (BMI) of less than 25 kg/m^2^. CI, confidence interval; M‐H, Mantel−Haenszel.

As shown from the characteristics in Table 1, eligible RCTs that reported primary outcomes involved various types of colonoscopies, different clinical settings and doses of PEG‐based solutions, and diverse patients with different ages and BMI values. Therefore, we did further subgroup analyses so as to eliminate the impact of these factors. Subgroup analyses still revealed a significant effect of OSS in the increase of PDR (Fig. 4) and ADR (Fig. 5) during colonoscopy after eliminating the impact of types of patients (outpatients), types of colonoscopies (morning colonoscopy), doses of bowel preparation solution (2‐L bowel preparation protocol), and mean age and BMI (patients with mean age >55 years and BMI <25 kg/m^2^), indicating the robustness of the pooled results.

### Secondary outcomes

**Figure 6:**
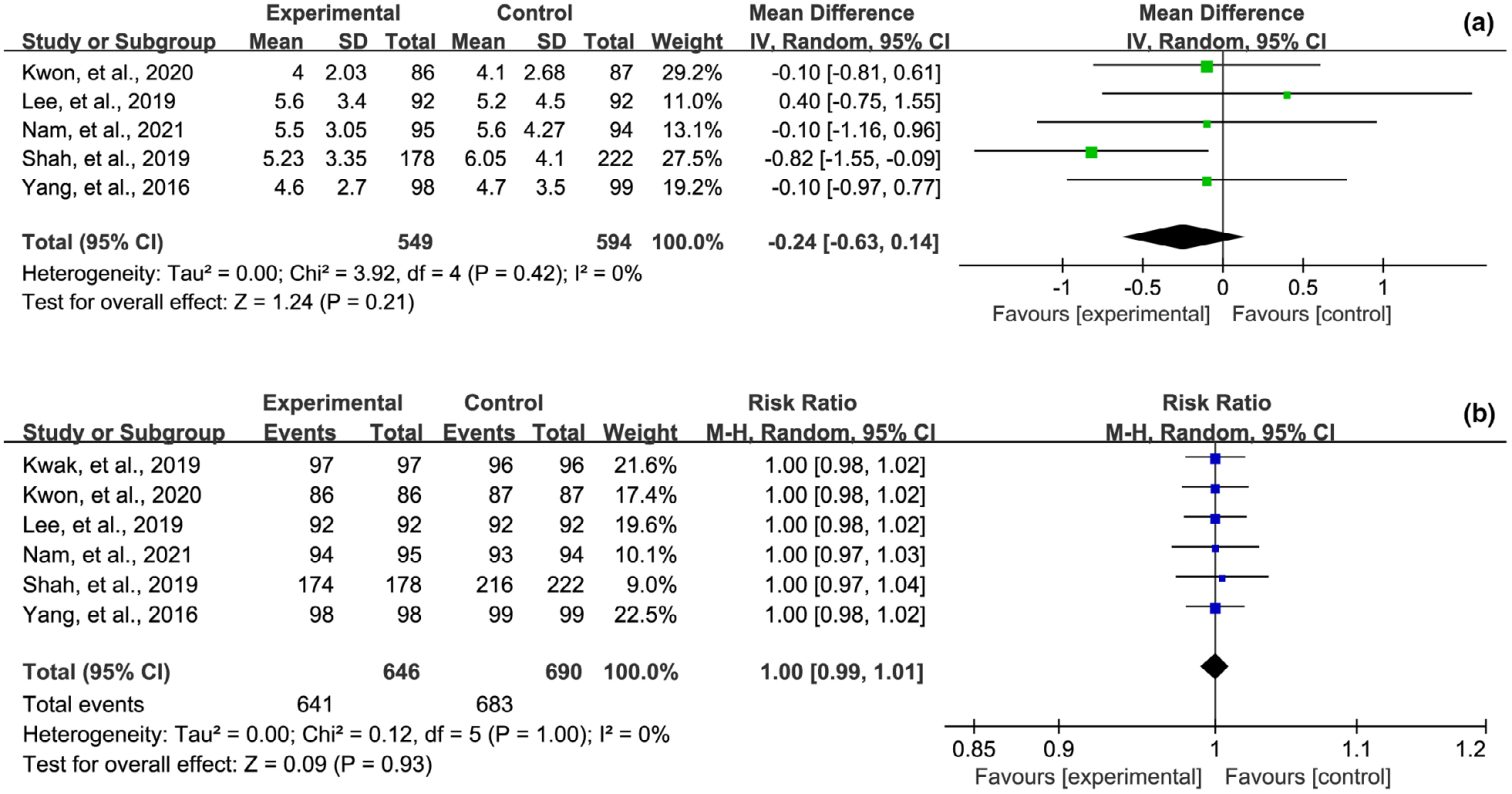
Meta‐analysis of the effect of oral sulfate solution on cecal insertion time (a) and cecal intubation rate (b). CI, confidence interval; IV, weighted mean difference; M‐H, Mantel−Haenszel.

**Figure 7:**
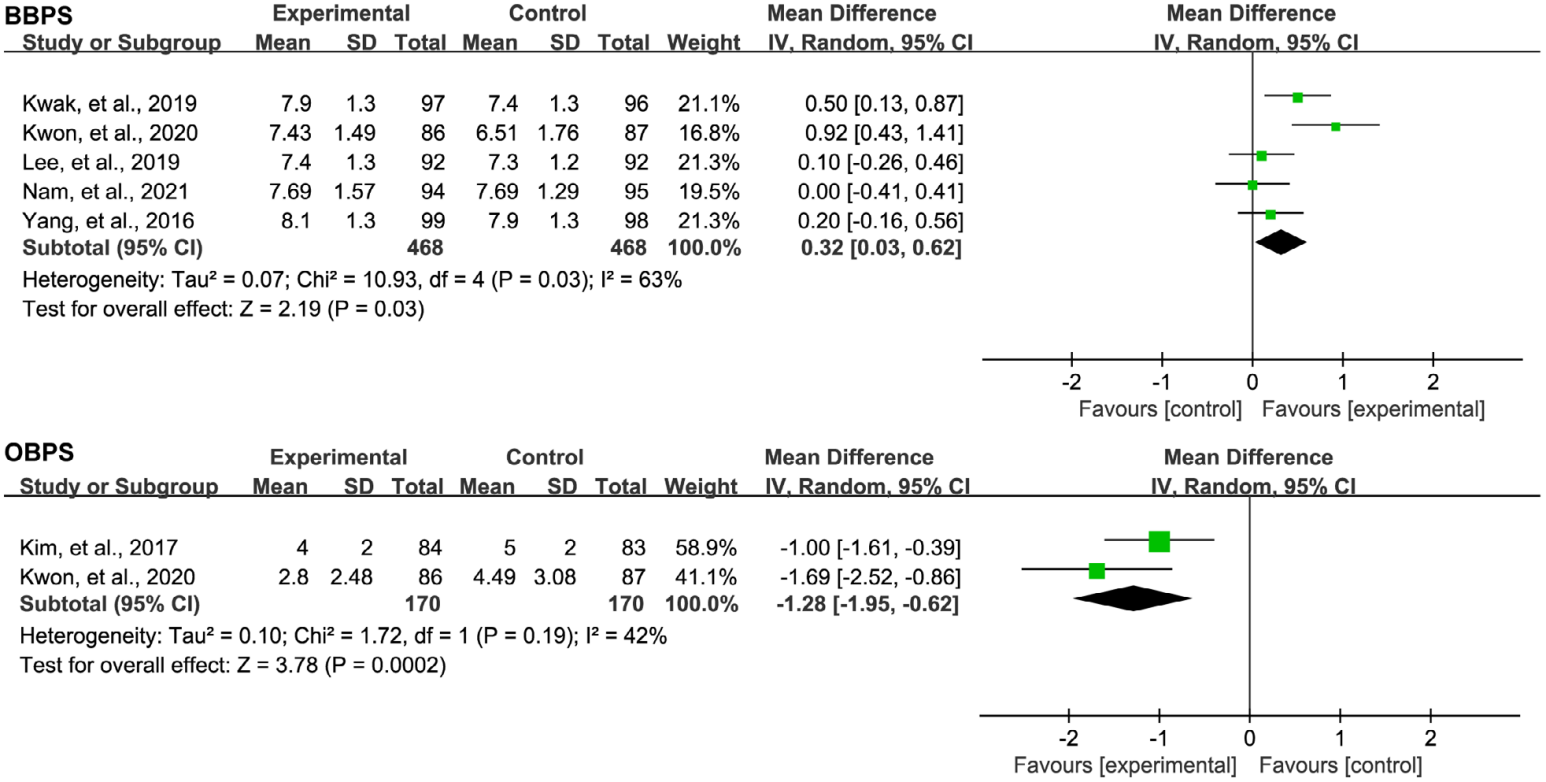
Meta‐analysis of the effect of oral sulfate solution on bowel preparation score according to Boston Bowel Preparation Scale (BBPS) or Ottawa Bowel Preparation Scale (OBPS). CI, confidence interval; IV, weighted mean difference.

Among the included eight RCTs, five^45^, ^46^, ^47^, ^48^, ^49^ reported data of CIT. Pooled analysis suggested a numerically shorter time to cecal intubation patients receiving OSS, although the difference was not statistically significant (MD −0.24, 95% CI −0.63−0.14, *P* = 0.21), as shown in Figure 6a. Six RCTs reported data of CIR, and pooled analysis suggested comparable results between OSS and PEG‐based solutions (RR 1.00, 95% CI 0.99−1.01, *P* = 0.93), as shown in Figure 6b. Five^44^, ^45^, ^46^, ^47^, ^49^ and two^20^, ^45^ RCTs reported BBPS and OPBS of colonoscopy, respectively. Pooled analyses suggested that, compared with the PEG‐based solutions group, the BBPS in the OSS group was significantly high (MD 0.32, 95% CI 0.03–0.62, *P* = 0.03) and the OBPS in the OSS group was significantly low (MD −1.28, 95% CI −1.95–0.62, *P* < 0.001), as shown in Figure 7, demonstrating that the quality of bowel preparation in the OSS group is better than that of PEG‐based solutions group.

## Discussion

Low‐volume bowel preparation bowel regimens have been developed and widely used for bowel cleansing before a colonoscopy in order to increase patients' compliance to bowel regimens. As a novel osmotic preparation, the efficacy and safety of OSS have been confirmed in clinical studies.^18^, ^19^ However, it is unclear whether OSS can ultimately increase PDR and ADR, although several studies have demonstrated that OSS has comparable or better quality of bowl preparation than PEG formulations and has acceptable safety.^14^, ^22^, ^23^ In this meta‐analysis, by pooling the eight RCTs, we first revealed that OSS for bowel preparation was associated with a significantly increased PDR and ADR during colonoscopy, which suggested that OSS might be an efficacious regimen for patients scheduled for a colonoscopy. Additionally, from the result of the analysis of the bowel preparation score, it was suggested that OSS significantly improved bowel preparation. Moreover, the results of analysis of CIT and CIR showed that OSS did not have a significant impact on the time to cecal intubation and successful CIR.

Theoretically, excellent bowel preparation should be associated with the increased detection rate of colorectal lesions, because better bowel preparation makes it possible to detect smaller polyps and adenomas. According to the results of a previous meta‐analysis,^23^ OSS would increase the detection of polyps and adenomas because it significantly increased the proportion of patients with excellent bowel preparation. However, among the included six RCTs that reported primary outcomes, only one found a significantly increased PDR^20^ or ADR^47^ in the OSS group. The other included RCTs did not detect differences between OSS and PEG‐based regimens in terms of PDR or ADR, which were consistent with the results of a retrospective study^41^ and a conference abstract.^50^ It was noted that the detected numbers of polyps and adenomas in patients receiving the OSS regimen were numerically higher than that in patients receiving PEG‐based regimens, although the statistical differences in terms of PDR and ADR were not achieved in the majority of included RCTs. We therefore speculate that a relatively insufficient sample size may be the major contributor to these inconsistent results.

It has always been known that meta‐analysis has the ability of increasing the statistical power through accumulating more sample sizes.^30^ In this study, we performed a meta‐analysis to investigate the overall effect of OSS for bowel preparation on PDR and ADR during colonoscopy. In the case of OSS for PDR and ADR, our meta‐analysis summarized all of the relevant data from eligible RCTs, substantially decreased the type II statistical error, and detected a significant effect of an OSS regimen on increasing PDR and ADR. Meanwhile, we performed several subgroup analyses to further confirm the robustness of pooled results through introducing several factors, including type of patients (outpatients vs. inpatients), mean age (>55 years vs. ≤55 years), mean BMI (<25 kg/m^2^ vs. ≥25 kg/m^2^), and type of colonoscopy (morning colonoscopy vs. afternoon colonoscopy). Certainly, we also found a higher score in BBPS and a lower score in OBPS in the OSS group, which suggests the main reason of OSS significantly increasing PDR and ADR during colonoscopy.

As quality indicators for colonoscopy, the associations between quality of bowel preparation, ADR, and CIR have been widely investigated.^51^ The present meta‐analysis suggested that patients consuming the OSS regimen might be more likely to achieve excellent bowel preparation (indicated as a higher BBPS score or lower OBPS score); however, the previously published meta‐analysis revealed a comparable rate in adequate bowel preparation between OSS and PEG + ASC regimens. Namely, procedural difficulty during colonoscopy examination, which is positively associated with CIT and CIR, was similar in patients consuming OSS or PEG regimens. Therefore, the differences in CIT and CIR between the two regimens did not achieve statistical significance, although those patients receiving the OSS regimen experienced numerically less CIT. Meanwhile, studies have suggested that insufficient bowel preparation is associated with a reduced PDR and ADR,^52^ as well as increased medical costs.^12^ In other words, excellent bowel preparation can increase the detection of polyps and adenomas, and then decrease medical expenditures. In 2016, Huynh *et al*.^38^ used a cost‐analysis model to examine cost savings associated with OSS related to PEG‐based regimens, and found that the use of OSS as the bowel cleansing agent before colonoscopy resulted in potential cost savings compared with PEG‐based regimens. From the cost savings perspective, the use of OSS as the cleansing agent is also considered to potentially increase detection of polyps and adenomas during colonoscopy.

An OSS regimen was associated with an increased excellent bowel preparation because the reduced volume may be more acceptable and tolerable;^48^ however, a previous meta‐analysis revealed a higher risk of nausea and vomiting in patients consuming the OSS regimen.^23^ Currently, the definitive reasons for this result remain unknown; however, an unpleasant taste and flavor caused by the sulfate component has been suggested as a possible reason.^20^, ^21^ Trial sequential analysis,^53^ which was conducted by TSA software 0.9.5.10 Beta (Copenhagen Trial Unit, Centre for Clinical Intervention Research, Rigshospitalet, Copenhagen, Denmark, https://www.ctu.dk/tsa), was used to further examine whether a definitive conclusion could be obtained for the risk of nausea and vomiting based on the results of a previous meta‐analysis.^23^ As shown in Figure S2, the Z‐curve for nausea crossed through the TSA‐adjusted monitoring boundary, although accumulated sample size was less than the required information size of 2039, and accumulated sample size for vomiting was more than the required information size of 605, although it did not cross through the TSA‐adjusted monitoring boundary, indicating that future studies could not change the conclusion. Therefore, as recommended by a previous meta‐analysis, the OSS regimen should not be prescribed for populations already predisposed to nausea and vomiting and patients with diabetes, gastroparesis, and/or foregut functional diseases.^23^

We must acknowledge some limitations in this meta‐analysis. First and foremost, this meta‐analysis may introduce some bias because it was performed on the basis of published study‐level data rather than published patient‐level data.^54^ Second, we performed subgroup analysis to investigate whether the type of patient, type of colonoscopy, volume of PEG solutions, and mean age BMI of patients will negatively affect the robustness of pooled results. However, sensitivity analysis could not be conducted to eliminate the influence of other factors on the pooled results. Third, we could not perform subgroup analysis to eliminate the impact of indications for colonoscopy on pooled results due to the complex data structure. Fourth, the heterogeneity examination suggested no evidence of statistical heterogeneity among a meta‐analyses of primary outcomes; it may be better to investigate clinical heterogeneity in this meta‐analysis rather than use it as a reason for not conducting one.^55^ Fifth, our meta‐analysis suggested that OSS may be a better bowel cleansing agent for increasing PDR and ADR; however, the impact of procedure‐related factors and endoscopist‐related factors should also be emphasized when considering our findings. In spite of the limitations explained above, the overall methodological quality of six RCTs that reported primary outcomes is good, and the accumulated sample size was 1693 (826 in the OSS group and 867 in the PEG‐based group), with a considerably decreased type II statistical error. Therefore, it is rational to suppose that our meta‐analysis provides clear evidence to answer the clinical question of the effect of OSS for bowel preparation on increasing PDR and ADR during colonoscopy.

## Conclusion

In summary, the present meta‐analysis provides relevant evidence that OSS for bowel preparation can increase the PDR and ADR through improving bowel preparation quality during colonoscopy. Therefore, the OSS regimen may be a promising low‐volume preparation alternative strategy for bowel preparation before colonoscopy.

## Conflict of Interest

Authors declare no conflict of interest for this article.

## Funding Information

None.

## Supporting information


**Figure S1** Funnel plot for polyp detection rate (PDR) (A) and adenoma detection rate (ADR) (B).
**Figure S2** Trial sequential analysis of nausea and vomiting.
**Table S1** Search strategies.
