# Supplementary material for: Oral sulfate solution benefits polyp and adenoma detection during colonoscopy: Meta‐analysis of randomized controlled trials
Source: Dig Endosc. 2022 Apr 6;34(6):1121–33. doi: 10.1111/den.14299 (PMC9545996; doi:10.1111/den.14299)
Supplement: Supplementary file 1 — Figure S1 Funnel plot for polyp detection rate (PDR) (A) and adenoma detection rate (ADR) (B). Figure S2 Trial sequential analysis of nausea and vomiting. Table S1 Search strategies. [file DEN-34-1121-s001.docx]

**Table S1. Search strategy**

a. PubMed

| NO. | Query | Results |
| --- | --- | --- |
| 8 | ((("Polyethylene Glycols"[Mesh]) OR ((((((((((((Polyethylene Glycol[Title/Abstract]) OR (Polyethylene Glycols[Title/Abstract])) OR (Macrogol[Title/Abstract])) OR (Macrogols[Title/Abstract])) OR (Polyethylene Oxide[Title/Abstract])) OR (Polyethylene Oxides[Title/Abstract])) OR (Polyethyleneoxide[Title/Abstract])) OR (Polyethyleneoxides[Title/Abstract])) OR (Polyoxyethylenes[Title/Abstract])) OR (Polyoxyethylene[Title/Abstract])) OR (Polyglycol[Title/Abstract])) OR (Polyglycols[Title/Abstract]))) AND ((oral sulfate solution[Title/Abstract]) OR (OSS[Title/Abstract]))) AND (("Colonoscopy"[Mesh]) OR (((Colonoscopy[Title/Abstract]) OR (Colonoscopies[Title/Abstract])) OR (Colonoscopic[Title/Abstract]))) | 25 |
| 7 | ("Colonoscopy"[Mesh]) OR (((Colonoscopy[Title/Abstract]) OR (Colonoscopies[Title/Abstract])) OR (Colonoscopic[Title/Abstract])) | 47,904 |
| 6 | ((Colonoscopy[Title/Abstract]) OR (Colonoscopies[Title/Abstract])) OR (Colonoscopic[Title/Abstract]) | 32,669 |
| 5 | "Colonoscopy"[Mesh] | 32,483 |
| 4 | (oral sulfate solution[Title/Abstract]) OR (OSS[Title/Abstract]) | 2,356 |
| 3 | ("Polyethylene Glycols"[Mesh]) OR ((((((((((((Polyethylene Glycol[Title/Abstract]) OR (Polyethylene Glycols[Title/Abstract])) OR (Macrogol[Title/Abstract])) OR (Macrogols[Title/Abstract])) OR (Polyethylene Oxide[Title/Abstract])) OR (Polyethylene Oxides[Title/Abstract])) OR (Polyethyleneoxide[Title/Abstract])) OR (Polyethyleneoxides[Title/Abstract])) OR (Polyoxyethylenes[Title/Abstract])) OR (Polyoxyethylene[Title/Abstract])) OR (Polyglycol[Title/Abstract])) OR (Polyglycols[Title/Abstract])) | 92,571 |
| 2 | (((((((((((Polyethylene Glycol[Title/Abstract]) OR (Polyethylene Glycols[Title/Abstract])) OR (Macrogol[Title/Abstract])) OR (Macrogols[Title/Abstract])) OR (Polyethylene Oxide[Title/Abstract])) OR (Polyethylene Oxides[Title/Abstract])) OR (Polyethyleneoxide[Title/Abstract])) OR (Polyethyleneoxides[Title/Abstract])) OR (Polyoxyethylenes[Title/Abstract])) OR (Polyoxyethylene[Title/Abstract])) OR (Polyglycol[Title/Abstract])) OR (Polyglycols[Title/Abstract]) | 32,765 |
| 1 | "Polyethylene Glycols"[Mesh] | 74,743 |

b. Embase

Session Results

.......................................................

No. Query Results Results Date

#8. #3 AND #4 AND #7 66 3 Nov 2021

#7. #5 OR #6 98,331 3 Nov 2021

#6. 'colonoscopy'/exp 89,344 3 Nov 2021

#5. colonoscopy:ti,ab,kw OR colonoscopies:ti,ab,kw OR 65,138 3 Nov 2021

colonoscopic:ti,ab,kw

#4. 'oral sulfate solution':ti,ab,kw OR 'oral sulfate 3,091 3 Nov 2021

solutions':ti,ab,kw OR oss:ti,ab,kw

#3. #1 OR #2 50,206 3 Nov 2021

#2. 'macrogol derivative'/exp 13,757 3 Nov 2021

#1. 'polyethylene glycol[':ti,ab,kw OR 'polyethylene 39,902 3 Nov 2021

glycols':ti,ab,kw OR macrogol:ti,ab,kw OR

macrogols:ti,ab,kw OR 'polyethylene

oxide':ti,ab,kw OR 'polyethylene oxides':ti,ab,kw

OR polyethyleneoxide:ti,ab,kw OR

polyethyleneoxides:ti,ab,kw OR

polyoxyethylenes:ti,ab,kw OR

polyoxyethylene:ti,ab,kw OR polyglycol:ti,ab,kw

OR polyglycols:ti,ab,kw

.......................................................

c. Cochrane library

ID Search Hits

#1 (Polyethylene Glycol):ti,ab,kw OR (Polyethylene Glycols):ti,ab,kw OR (Macrogol):ti,ab,kw OR (Macrogols):ti,ab,kw OR (Polyethylene Oxide):ti,ab,kw 4364

#2 (Polyethylene Oxides):ti,ab,kw OR (Polyethyleneoxide):ti,ab,kw OR (Polyethyleneoxides):ti,ab,kw OR (Polyoxyethylenes):ti,ab,kw OR (Polyoxyethylene):ti,ab,kw 32

#3 (Polyglycol):ti,ab,kw OR (Polyglycols):ti,ab,kw 0

#4 #1 or #2 or #3 4387

#5 MeSH descriptor: [Polyethylene Glycols] explode all trees 3048

#6 #4 or #5 4839

#7 (oral sulfate solution):ti,ab,kw OR (oral sulfate solutions):ti,ab,kw OR (OSS):ti,ab,kw 922

#8 (Colonoscopy):ti,ab,kw OR (Colonoscopies):ti,ab,kw OR (Colonoscopic):ti,ab,kw 7380

#9 MeSH descriptor: [Colonoscopy] explode all trees 2158

#10 #8 or #9 7578

#11 #6 and #7 and #10 69


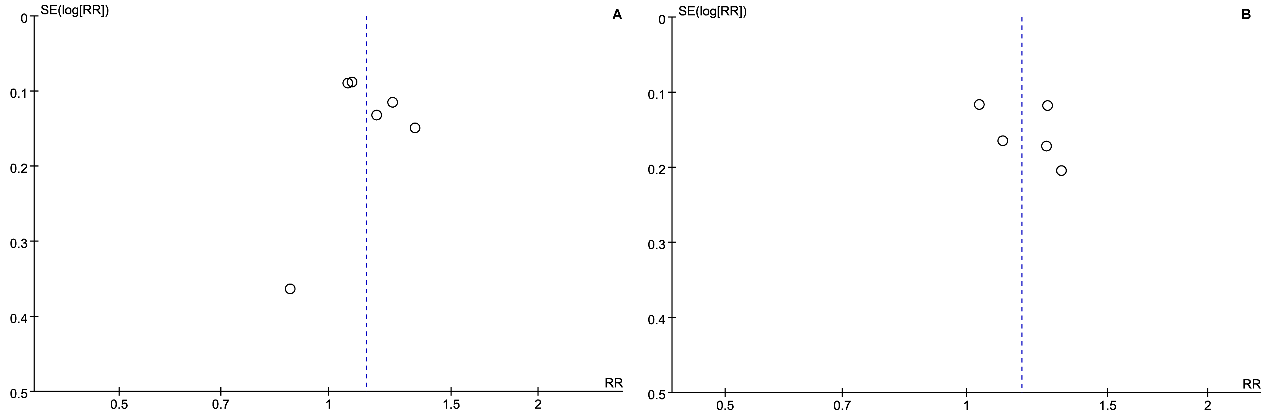


**Figure S1.** Funnel plot for polyp detection rate (PDR) (A) and adenoma detection rate (ADR) (B).


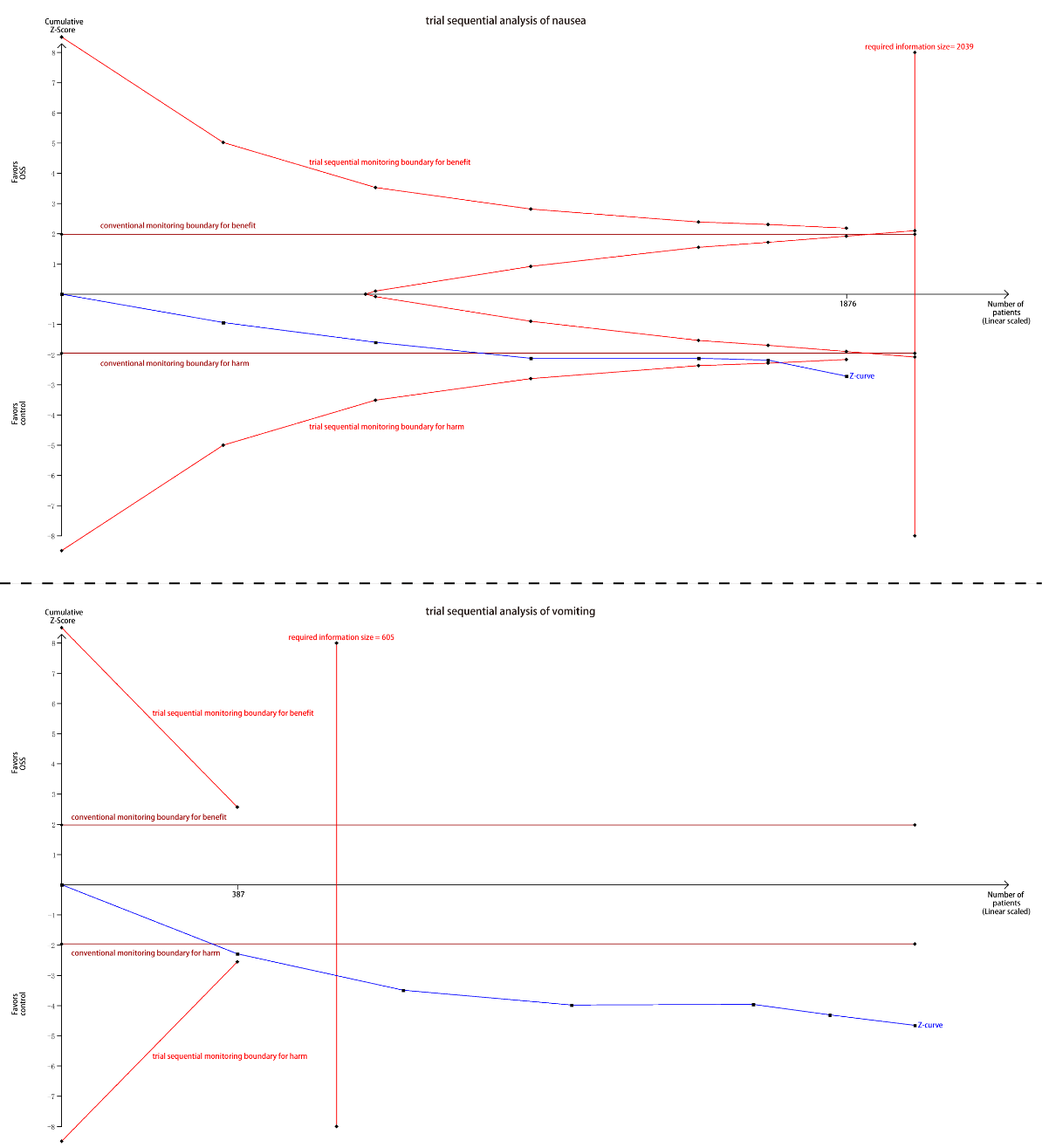


**Figure S2.** Trial sequential analysis of nausea and vomiting. The data of nausea and vomiting was extracted from the previously published meta-analysis. We calculated required information sizes through introducing type I error of 5%, statistical power of 80%, empirical incidences in research and control groups (calculated from previous meta-analysis), and diversity-adjusted heterogeneity (inserted by software automatically).
